# Fly-by-Pi: Open source closed-loop control for geotechnical centrifuge testing applications

**DOI:** 10.1016/j.ohx.2020.e00151

**Published:** 2020-10-17

**Authors:** André Broekman, Schalk Willem Jacobsz, Hendrik Louw, Elsabé Kearsley, Tiago Gaspar, Talia Simone Da Silva Burke

**Affiliations:** Department of Civil Engineering, University of Pretoria, Pretoria, South Africa

**Keywords:** Controller, Cave mining, Piles, Soil-structure interaction, Physical modelling, Raspberry Pi, Python

## Abstract

Geotechnical centrifuges are valuable instruments for physical modelling of complex geotechnical problems in a controlled laboratory setting. In comparison to full-scale testing, scaled models are cost effective to construct and instrument and, when tested in a geotechnical centrifuge at increased centrifugal accelerations, are capable of replicating full-scale stress–strain soil behaviour. Centrifuge modellers require specialised hardware and instruments capable of functioning under high accelerations. Such hardware is costly, nearly always purpose built, and often rely on commercial, closed-source data acquisition systems, hardware and control systems.

This paper demonstrates a novel and versatile, low cost, open source logger and control system that works in parallel alongside existing centrifuge hardware. This solution, termed Fly-by-Pi, was developed using the Raspberry Pi microcomputer. The system provides closed-loop control of linear actuators with the ability to operate in either cyclic, monotonic, or static load- or displacement-control. The control mechanism can be reprogrammed according to experimental requirements, even during flight in the centrifuge. Three independent experiments are described which included the Fly-by-Pi controller as a key component in their operation. Based on the experience gained during these experiments, the authors encourage wide-spread adoption of open-sourced hardware solutions in extreme testing environments.

## Highlights

• Successful open source hardware integration in a geotechnical centrifuge.
• Lateral loading and block cave mining controller in a geotechnical centrifuge.
• Monotonic and cyclic loading controller in a geotechnical centrifuge.
• Raspberry Pi integration for civil engineering physical model studies.

## Graphical abstract

**Figure:**
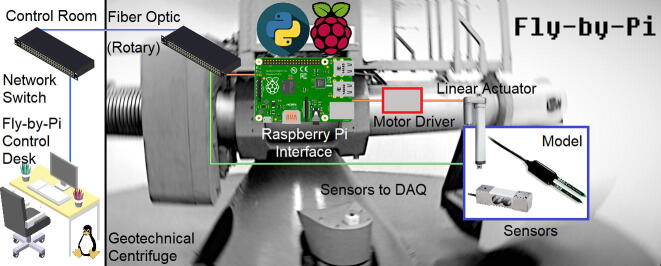

## 1 Specifications table

**Table:** 

Hardware name	Fly-by-Pi
Subject area	Engineering and Material Science
Hardware type	Measuring physical properties and in-lab sensors Electrical engineering and computer science
Open Source License	Creative Commons Attribution-ShareAlike
Cost of Hardware	$279.13 (including one linear actuator)
Source File Repository	https://doi.org/10.17605/OSF.IO/75H3A

## 2 Hardware in context

Geotechnical centrifuges are sophisticated physical modelling instruments available to study geotechnical problems. By increasing the acceleration field on a scale model using a geotechnical centrifuge, the stresses and resulting strains in the small-scale model are increased to match that of the full-scale equivalent [1]. Non-linear behaviour of rock and soil, which normally exhibit stress histories, complex interactions with moisture and particle size distributions of the granular media, necessitates the use of model studies. Despite advances in numerical modelling, physical models serve as an external calibration measure to validate numerical results especially for experiments that present complex interaction phenomena not suited for current software applications. Active areas of research interest pursued at the University of Pretoria include modelling of pipe deflection as a function of sinkhole development [2], cavity propagation [3] in sinkhole and cave mining applications [4], small strain stiffness measurements [5], cyclic loading of railway embankments [6] and ultra-thin continuously reinforced concrete pavements (UTCRCP) [7].

Despite presenting several advantages over constructing full scale models, centrifuge modelling poses several practical challenges in performing experiments. Any external action, whether the addition or removal of fluids, application of (cyclic) force(s) on the soil/structure, or the transfer and movement of material, requires a sophisticated and robust array of actuators, data acquisition and control systems, linking the inaccessible centrifuge chamber and control room. Data signals from the sensors are amplified, sampled at a high frequency and transmitted to the control room for storage and analysis. The actuators that perform the desired actions on the model in-flight range from simple pneumatic, hydraulic or electric types, manually activated from the control room, to sophisticated robotic systems [8] with interchangeable tools that can perform activities such as soil nailing, pile driving, tunnelling and soil excavation [9]. By virtue of the complexity and specialised nature of centrifuge testing, the cost and operational complexity of acquiring, installing, calibrating and operating control systems for experiments are often prohibitive when considering the required level customisability desired. Different sub-components of control systems typically rely on proprietary, closed-sourced software and interfaces to operate correctly, without the option to add third party hardware and software solutions.

As the complexity and duration of experiments have increased during the last few years, so has the demand for customisable, low-cost and programmable actuators and control systems [7], [9]. In the context of open-source hardware and software solutions, augmented by the proliferation and wide availability of powerful, low-cost microcontroller and microprocessor platforms, such a solution can be readily engineered. These solutions do not aim to replace more specialised commercial hardware solutions; instead, it addresses a significant proportion of instrumentation requirements for projects that are “deemed to satisfy”. This approach is aligned with the Civiltronics framework [11], [12], [13], whereby knowledge of complementary science, technology, engineering and mathematics (STEM) disciplines such as computer science and electronic engineering are combined with traditional civil engineering expertise to develop innovative solutions.

This paper presents a novel, closed-loop, open-source actuator control system for a 150 g-ton geotechnical centrifuge [14], located at the University of Pretoria in South Africa. The system can be customised and programmed for each experiment’s requirements and modified in-flight during testing. The Fly-by-Pi controller, using a solid-state Raspberry Pi single-board computer at its core, is inspired by the versatility of fly-by-wire control systems of modern aircrafts. Closed-loop control is achieved through sensor feedback from both the actuator and the instrumented model that are communicated via the existing centrifuge data acquisition system to the Fly-by-Pi controller. This is performed without the need for any alterations to the existing centrifuge data acquisition and control systems.

## 3 Hardware description

**Fig. 1:**
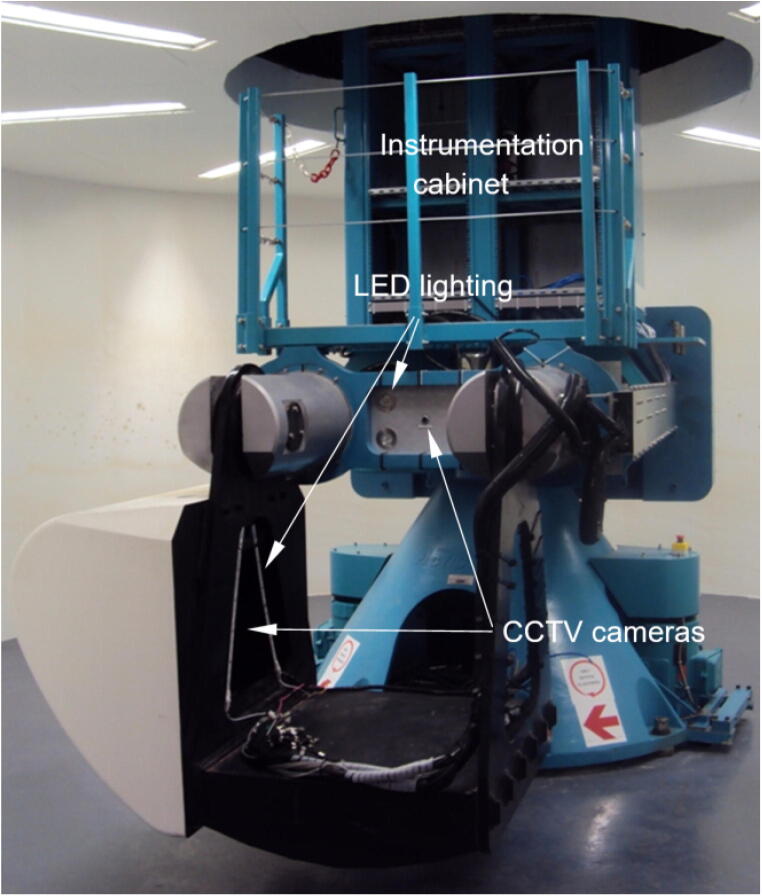
The 150 g-ton centrifuge at the University of Pretoria [14]

An Actidyn C67-4 geotechnical centrifuge (Fig. 1) was commissioned by the University of Pretoria in 2012 [14]. The 150*g*-ton centrifuge has a radius of 3 m and is capable of testing models weighing up to one metric ton up to 150 g, rotating at a maximum speed exceeding 230 km/h.

Several recent research projects required combinations of load and displacement controlled cyclic loading to be applied to models using off-the-shelf electric linear actuators. Commercial solutions for entry level universal control systems are available but are costly and typically financially unfeasible, particularly for smaller research groups. The alternative is manual operation from the control room, but this is not practical when a large number of load cycles has to be applied.

A cost-effective control system was required for three research projects executed in parallel. The first two projects investigated cyclic loading on scaled single piles in sand [15] and clay, with the third modelling block cave mining operations [4]. The first project required a single linear actuator applying cyclic lateral loads to piles, while the second project replicated the removal of supports from a model rock mass using five linear actuators. The design requirements for the control system suitable for both projects are summarised below:

• Remote control of single or multiple linear actuator(s).
• Support for multiple monotonic, static or cyclic (load and displacement) control mechanisms.
• Closed-loop control using feedback from the instrumented model in-flight.
• Reliable and continuous operation in an up to 80-g operating environment for a minimum of 72 h.
• Simple integration into the existing centrifuge infrastructure without significant modifications.
• Real-time, wired communication using ethernet (IEEE 802.3).
• Optional data acquisition and storage.

Based on the list of requirements, research was carried out to identify the most cost-effective solution based on locally available hardware platforms and capabilities. Owing to the need for high-speed and reliable communications, together with the ability to program the control system in-flight, the Raspberry Pi platform was adopted. The Raspberry Pi offers a small physical footprint, completely solid-state architecture with no active cooling requirements and a wide support community to accelerate the development, prototyping and testing phases. Raspberry Pi microcomputers have been used in a diverse range of applications ranging from agriculture [16], environmental sciences [17] and medicine [18]. Raspberry Pis are deployed aboard the international space station (ISS) to advance student participation in the sciences [19] and in the oceans to monitor aquatic life [20]. Their demonstrated versatility and robustness lend their application to adverse operating environments. Adopting an open source development workflow allows for modification of the hardware and software for other future projects by civil engineering students. Such modifications are possible without the need for extensive knowledge and experience in either electronic engineering or computer science. In the event of hardware failure, components are readily available through local vendors and distributors at a low cost compared to a commercially available alternative. The Fly-by-Pi controller is best described as a hybrid between a dedicated control system and a flexible logger, offering the benefits of both intelligently controlling actuators, whilst processing signals and storing data as required.

**Fig. 2:**
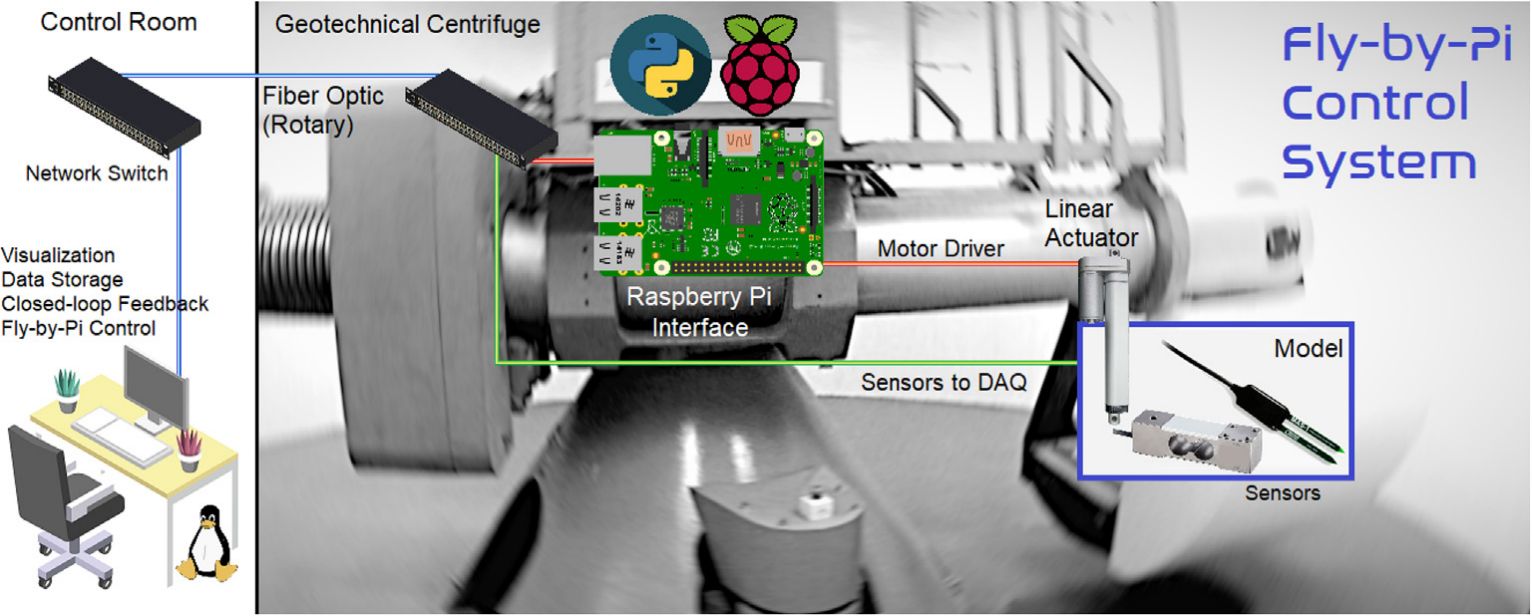
Graphical overview of the Fly-by-Pi controller.

The primary function of the Fly-by-Pi controller is to control a number of linear actuators at a specific speed and/or load in scaled geotechnical model studies (Fig. 2). To maintain control within a predefined envelope of either load or displacement, feedback from the primary centrifuge data acquisition system (DAQ), is required. Ideally this would be accomplished through IP-based network communication or serial communication protocols. Due to the closed source nature of the firmware, this was not a viable solution. Instead, the input signal received by the proprietary DAQ (with internal signal conditioning and amplification) was configured via software to be rerouted to the DAQ’s complimentary output port (BNC or DB15 connector). This duplicated and amplified signal was then interfaced directly with the Raspberry Pi’s dedicated high-speed, multi-channel analog-to-digital converter (ADC) to provide a direct interface from the primary DAQs. This configuration is cost-effective and allows any type of specialised transducer to be connected to the Fly-by-Pi controller via the DAQ without occupying ports on the existing system. The centrifuge DAQ system retains the responsibility of conditioning, amplifying, processing and storing data with a synchronised time signal, leaving the Raspberry Pi to only control the linear actuator(s).

**Fig. 3:**
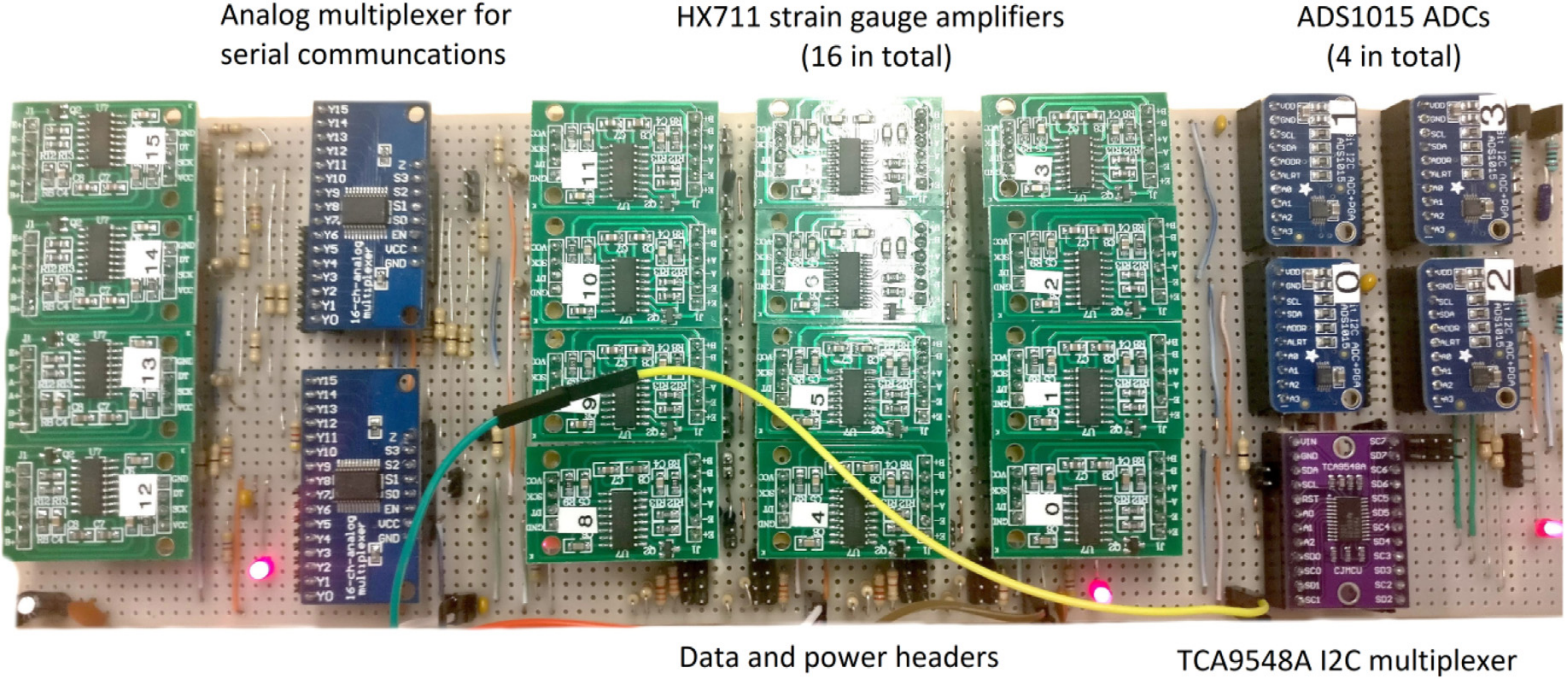
Amplifiers, multiplexers and ADCs installed onto a single Veroboard.

**Fig. 4:**
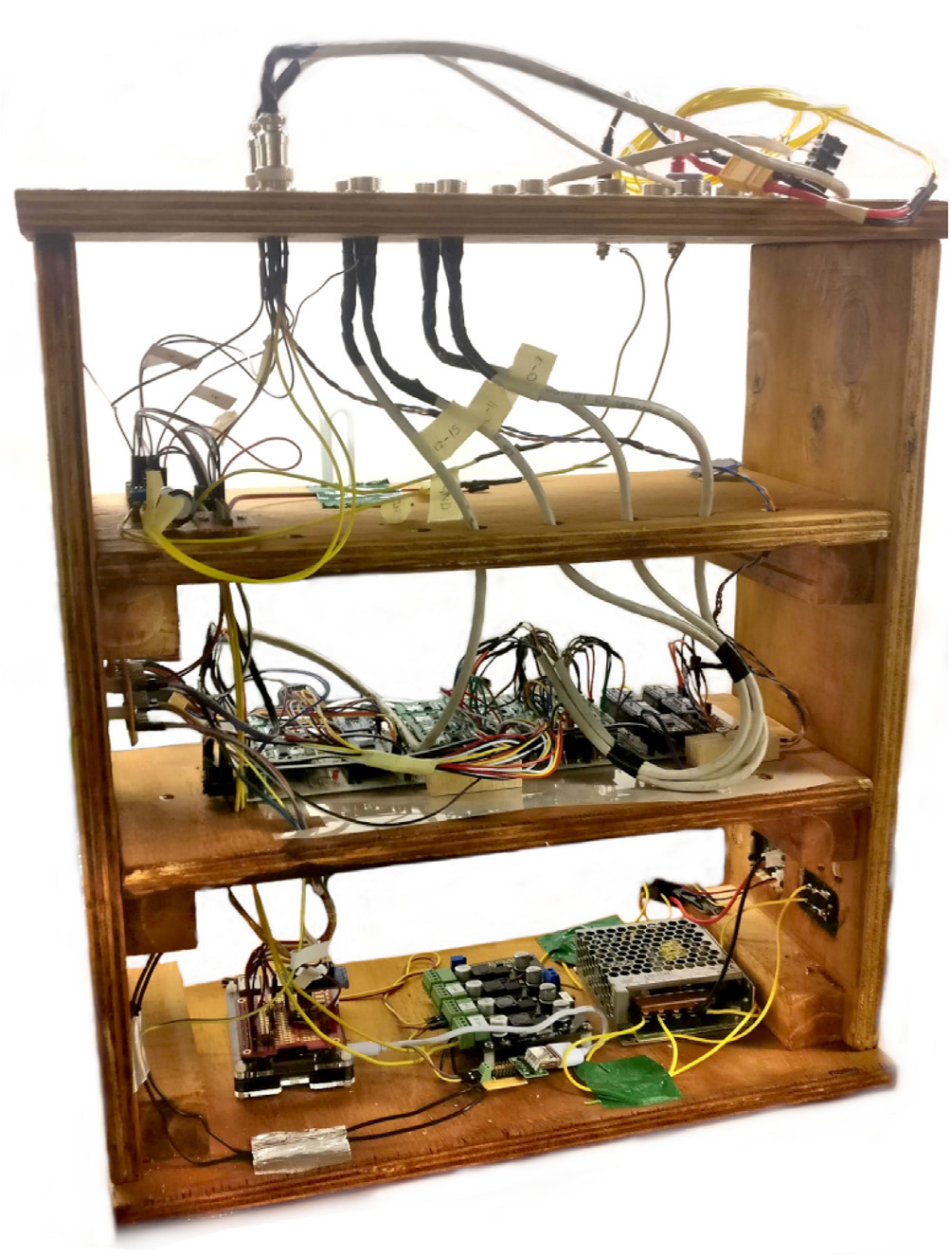
Early Fly-by-Pi prototype with all the electronic components.

The Fly-by-Pi controller demonstrates the following advancements that is applicable to the wider user community:

• Simple integration and automation of linear actuators and motors suitable for repetitive loading applications. For certain use cases, linear actuators can be substituded for stepper motors yielding significant cost savings.
• Demonstrating the potential of fusing together disjointed hardware and software platforms through the implemenation of a low-cost ADC.
• Generalisation of cyclic load and displacement control mechanisms that are applicable beyord the realm of geotechnical, for example fatigue and strength testing of different materials such as biological or 3D printed materials.
• Implementation of the ubiqtious Raspberry Pi controller system allowing for the integration into existing hardware solutions that are designed around the same ecosystem.

## 4 Design files

The complete list of files is summarised in Table 1. These files provide the necessary information and software to duplicate and implement an equivalent Fly-by-Pi controller. The files are freely accessible at the Open Science Framework source file repository linked together with this manuscript.

**Table 1:** Complete list of design files.

Design file name	File type	Open source license	Location of the file
LoadControl.py	Python script (.py)	CC BY 4.0	Source file repository (Source Code folder)
TimeControl.py	Python script (.py)	CC BY 4.0	Source file repository (Source Code folder)
StressTestADC.py	Python script (.py)	CC BY 4.0	Source file repository (Source Code folder)
mMotorDriver.py	Python script (.py)	CC BY 4.0	Source file repository (Source Code folder)
mMCP3434	Python script (.py)	CC BY 4.0	Source file repository (Source Code folder)
IndividualExperiments	Folder	CC BY 4.0	Source file repository (Source Code folder)
UDPDemo	Folder	CC BY 4.0	Source file repository (Source Code folder)
UserGuide.pdf	PDF	CC BY 4.0	Source file repository (User Guide folder)
FlyByPi_Controller_Layout.tif	Image	CC BY 4.0	Source file repository (Schematics folder)
FlyByPi_Schematic.pdf	PDF	CC BY 4.0	Source file repository (Schematics folder)
FlyByPi_Schematic.sch	ExpressSCH	CC BY 4.0	Source file repository (Schematics folder)
Media	Folder	CC BY 4.0	Source file repository

*LoadControl.py*: One of the primary control scripts written in Python. This executes the load control mechanism as described in Chapter 6. The individual control variables are defined within the script file.

*TimeControl.py*: One of the primary control scripts written in Python. This executes the time-based cyclic control mechanism as described in Chapter 6. The individual control variables are defined within the script file.

*StressTestADC.py*: A script written in Python to evaluate the data integrity of the ADC sensor readings. This can be used to test the performance and reliability of the ADC’s communications at different accelerations.

*mMotorDriver.py*: A generic class file that defines the motor driver instance with a few basic functions, such as changing the direction of the motor or configuring the duty cycle. Can be modified according to the specific motor driver utilised.

*mMCP3424.py*: Class file for the MCP3424 ADC sourced from the manufacturer’s website.

*IndividualExperiments*: A folder containing 3 individual experiment configurations as additional examples of the code implementation.

*UDPDemo*: A folder containing example Python scripts that can be used to send simple data string between a server and client. Potential for integration into more advanced systems to send and receive data between different nodes in a network configuration.

*UserGuide.pdf*: A detailed guide highlighting possible network configurations for the Fly-by-Pi and file management that tend to be overlooked, but is nonetheless of value to accelerate research and development for experiments.

**Fig. 5:**
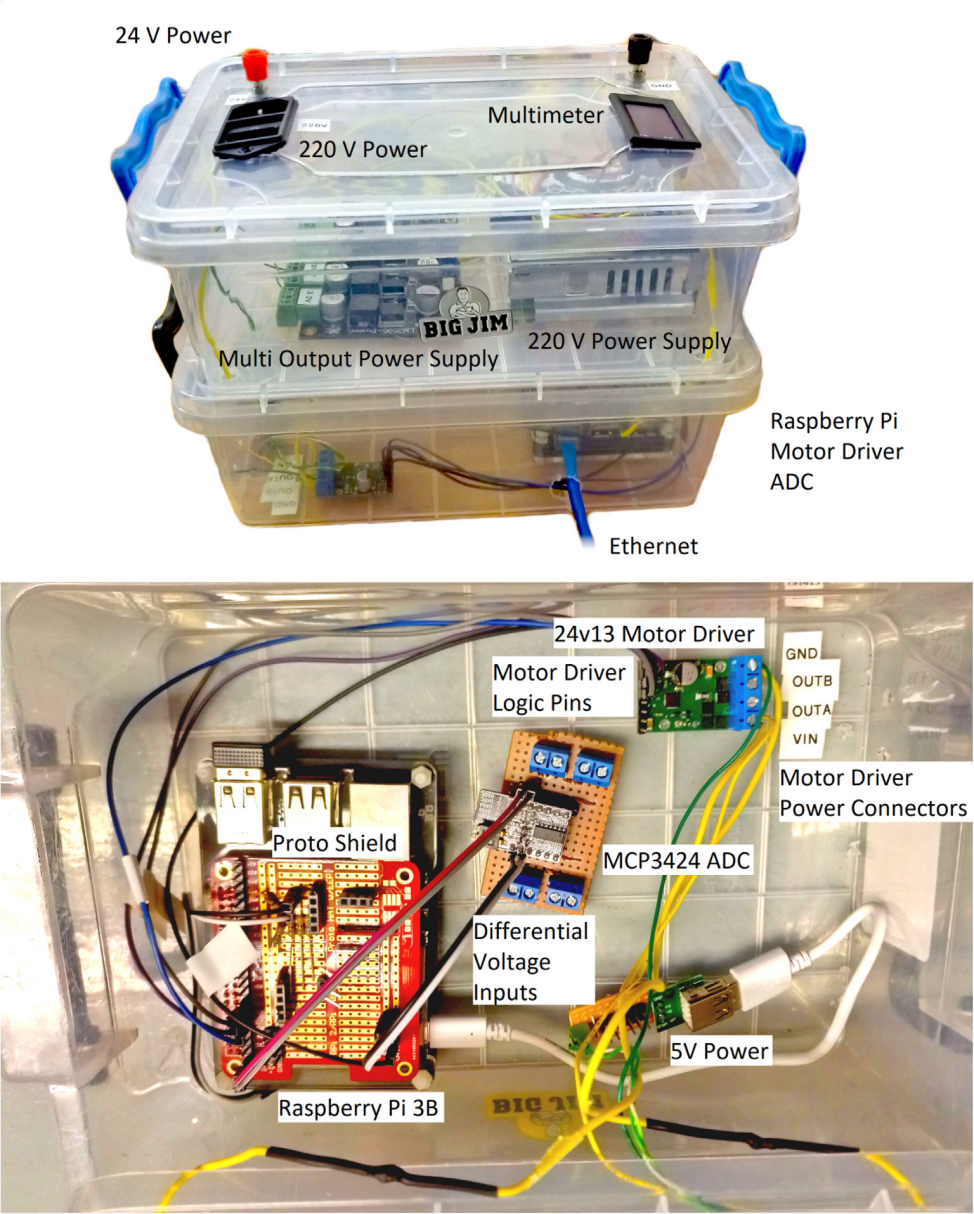
Fly-by-Pi controller installed within a plastic container.

*FlyByPi_Controller_Layout.tif*: Annotated photograph of the Fly-by-Pi hardware (identical to Fig. 5).

**Fig. 6:**
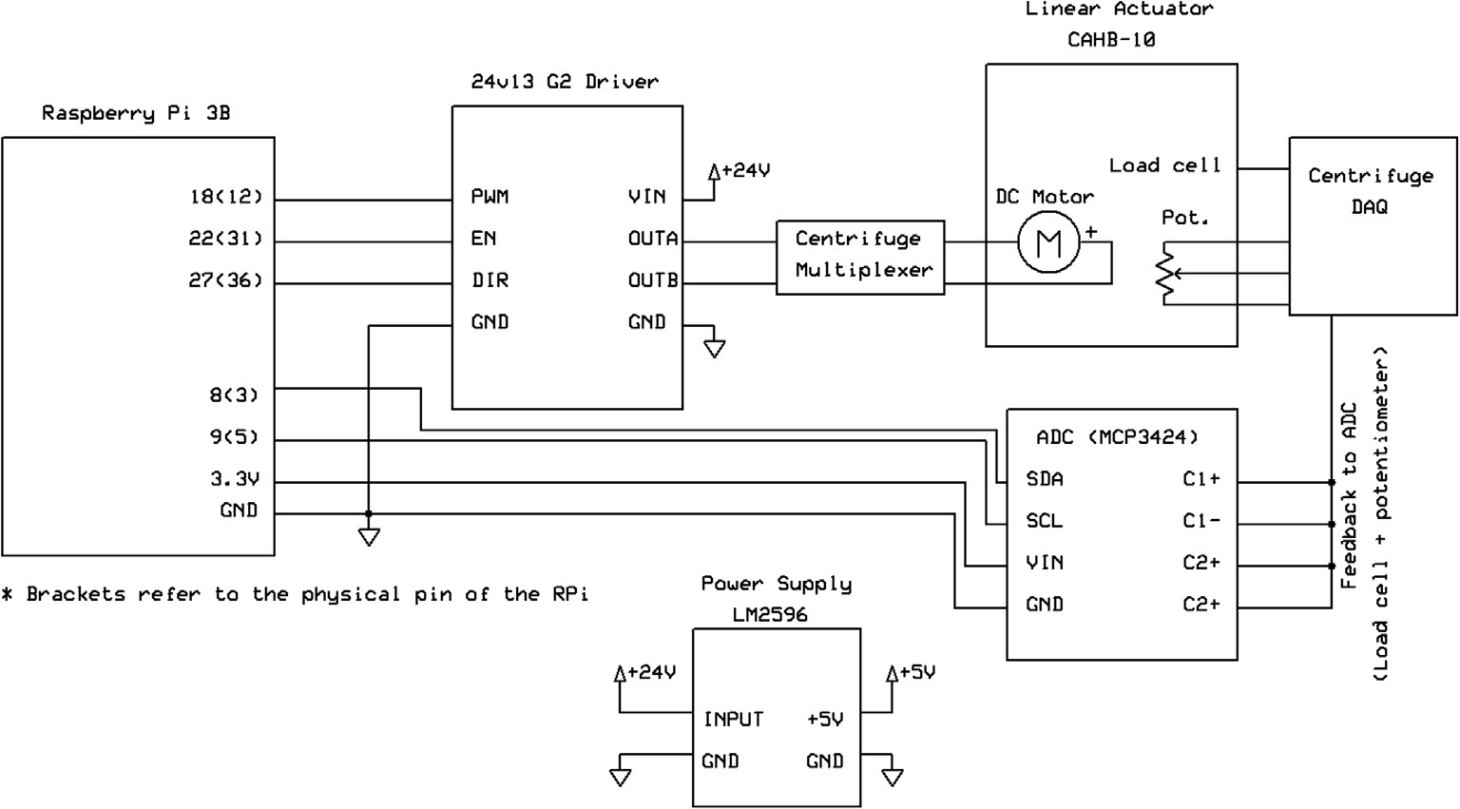
Electronic design of the Fly-by-Pi controller.

*FlyByPi_Schematic.pdf*: Electronic schematic detailing the hardware configuration of the Fly-by-Pi (identical to Fig. 6).

*FlyByPi_Schematic.sch*: ExpressSCH electronic schematic file detailing the hardware configuration of the Fly-by-Pi (identical to Fig. 6).

*Media*: Two additional photographs of the Fly-by-Pi controller in the centrifuge alongside a short video demonstrating the time-based cyclic control mechanism.

## 5 Bill of materials

The complete bill of materials (BOM) to replicate the Fly-by-Pi control system and linear actuator is listed in Table 2 and Table 3 respectively. For brevity, the prototype’s extensive BOM is not listed as only the current implementation of the Fly-by-Pi controller is relevant. The listed components are not specialised and can be sourced online from several local (South African) and international suppliers.

**Table 2:** Fly-by-Pi controller Bill of Materials.

Designator	Component	Number	Cost per unit – currency	Total cost – currency	Source of materials	Material type
Fly-by-Pi	Raspberry Pi (Model 3B)	1	$29.78 USD	$29.78 USD	PiShop	Other
	Enclosure (Pibow 3 Coupé Ninja)	1	$7.91 USD	$7.91 USD	PiShop	Other
	SD Card (16 Gb Class 10)	1	$6.69 USD	$6.69 USD	PiShop	Other
	Prototype shield	1	$5.39 USD	$5.39 USD	PiShop	Other
	Adhesive (double-sided adhesive square, 12 pack)	1	$3.96 USD	$3.96 USD		Other
	Power supply (LM2596 multi output)	1	$14.41 USD	$14.41 USD	MicroRobotics	Other
	USB Cable (micro-B, 1000 mm)	1	$2.72 USD	$2.72 USD	MicroRobotics	Other
	Ethernet cable (2 m UTP CAT5E)	1	$2.12 USD	$2.12 USD	MicroRobotics	Other
	USB Breakout (USB-A female)	1	$1.51 USD	$1.51 USD	MicroRobotics	Other
	BNC Connector (panel mount)	4	$1.46 USD	$5.87 USD	RS	Other
	BNC Connector (cable mount connector)	4	$2.77 USD	$11.07 USD	RS	Other
	Pinhead male (2.54 mm 40-pin breakaway straight male, 10 pack)	1	$0.91 USD	$0.91 USD	MicroRobotics	Other
	Pinhead female (2.54 mm 40-pin single-row female header, 4 pack)	1	$0.54 USD	$0.54 USD	MicroRobotics	Other
	Terminal blocks (screw, 5 mm, 10 pack)	1	$1.21 USD	$1.21 USD	MicroRobotics	Other
	Jumper wire (2.54 mm, 10 cm, 50 pack)	1	$4.78 USD	$4.78	MicroRobotics	Other
	Enclosure (generic 2 L transparent plastic with removable lid)	1	$1.05 USD	$1.05 USD		Other
	Cable ties (reusable, 200 mm)	4	$0.26 USD	$1.05 USD		Other
	Insulated wires (per m, 20 AWG)	4	$0.12 USD	$0.49 USD	MicroRobotics	Other
	Banana plugs (2 plugs)	1	$1.51 USD	$1.51 USD	RS	Other
	XT60 connector (to banana plug)	1	$2.72 USD	$2.72 USD	MicroRobotics	Other
	XT60 connector (male to female)	1	$2.05 USD	$2.05 USD	MicroRobotics	Other
	Veroboard (100x100 mm)	1	$1.51 USD	$1.51 USD	MicroRobotics	Other
Analog to digital converter (ADC)	ADC (MCP3424, DFRobot breakout)	1	$14.22 USD	$14.22 USD	MicroRobotics	Other
Motor driver	Motor Driver (high-power 24v13, Pololu)	1	$36.26 USD	$36.26 USD	MicroRobotics	Other
	Total cost per Fly-by-Pi controller			$171.71 USD

**Table 3:** Linear actuator bill of materials.

Designator	Component	Number	Cost per unit – currency	Total cost – currency	Source of materials	Material type
Linear actuator	Linear actuator Detail: CAHB-10	1	$100.12 USD	$100.12 USD	SKF	Other
	C13 connector cable	1	$7.30 USD	$7.30 USD	MicroRobotics	Other
	Total cost per linear actuator			$107.42 USD		

## 6 Building instructions

The generalised building instructions for duplicating the Fly-by-Pi controller is sub-divided into a short summary of each of the key components, the original prototype that failed and the current implementation. The detailed software configuration of the Raspberry Pi can be found in the corresponding data repository of this article; this includes basic network and IP address configuration and software installations. The most recent detailed installation guide of the Raspbian operating system can found on the website of the Raspberry Pi Foundation.

### 6.1 Linear actuator

The linear actuator used for the projects was the SKF CAHB-10 Series with a load capacity of 500 N and either a 50 mm or 100 mm stroke length respectively [21]. The actuator is powered by a 12 V/24 V/2A DC motor connected through a 30:1 ratio reduction gearbox. This actuator, with a unit price of approximately $120, is significantly more cost effective than a stepper motor. The actuator is equipped with an internal potentiometer, providing a linear output voltage in response to the actuator’s position. For load control during the cyclic loading applications a button load cell with a full-scale capacity of 500 N was attached to the end of the actuator to monitor the applied load. C13 connectors were added to each of the actuator’s motor connectors to ensure reliability in-flight.

### 6.2 Motor driver

The relatively modest power requirements of the linear actuator provide flexibility in the selection of an appropriate motor driver. Both the scale of the geotechnical models and the material properties of soils necessitated the need for precise motor control. The torque of the motor is significant compared to the resistance provided by, for example, the scaled model piles during the lateral cyclic load experiment. Motor torque was controlled by using pulse width modulation (PWM) to moderate the effective power supplied to the DC motor. The PWM frequency was fixed at 300 Hz with the duty cycle configured by the user as a percentage value, where 100% indicates full power. The high-power 24v13 G2 motor driver breakout from Pololu was selected for this project [22], supporting an excitation voltage range from 6.5 V to 40 V, a continuous driving current of 13 A, PWM and directional control. The dedicated PWM pin on the Raspberry Pi provides the required hardware interface for the motor driver. Initial testing showed that a 12 V power supply did not provide enough power, notably for tests in stiffer clay materials. A 24 V power supply was thus used for all subsequent experiments.

### 6.3 Analog to digital converter (ADC)

The 4-channel, 18-bit MCP3424 breakout board manufactured by DFRobot [23] was used as the primary interface between the Fly-by-Pi controller and the centrifuge’s control system. The ADC is designed for differential input signals that is ideal for directly connecting transducers such as Wheatstone bridges, if required. The Programmable Gain Amplifier (PGA) can be configured independently for each channel depending on the experimental design requirements to provide the maximum resolution. Communication over the I2C bus allows for faster sampling rates, error identification and expandability for additional devices. Both the load cell and potentiometer on the linear actuator were calibrated prior to the experiment. The calibration (volt per metric unit) factor for each was entered and stored in the control script for the experiment to convert the signal to calibrated metric values.

### 6.4 Prototyping

The Fly-by-Pi controller presented in the article is an optimised version of an initial experimental prototype. The original prototype consisted of 16 multiplexed, dual-channel differential strain amplifiers (HX711), 4 multiplexed general-purpose ADCs (ADS1015) from Adafruit [24] and the 24v13 G2 motor driver (Fig. 3). The first prototype is shown in Fig. 4. The unit was positioned adjacent to the centrifuge model for ease of connectivity with numerous strain gauges and the linear actuator. Electrical interference was identified as the primary cause of significant signal noise and data corruption during in-flight testing, with the severity proportional to the rotational velocity of the centrifuge. Such electrical noise is a problem often encountered during centrifuge testing at high accelerations. This affected communication for both the serial and I2C communication protocols. Attempts to provide insulation against electrical interference in the form aluminium foil covering the enclosure proved futile. In this configuration, it was not possible to share data with the actuator control system, limiting the value of such a system.

A standalone Raspberry Pi was successfully stress tested in the centrifuge at a maximum of 80-g to verify the reliability of hardware when subjected to extreme acceleration. The Fly-by-Pi controller was relocated to the centrifuge instrumentation cabinet (Fig. 1) and mounted on the rotation axis instead of directly on the swing platform, freeing up more space and substantially reducing the centrifugal acceleration experienced by the hardware. Using standard ping tests, no degradation of communication integrity over ethernet could be observed, despite the high intensity magnetic fields generated by the twin 120 kW motors powering the centrifuge.

### 6.5 Current implementation

The experience gained from the first protype led to the development of the improved Fly-by-Pi controller design currently used (Fig. 5). The low-cost amplifiers that were prone to noise were replaced in favour of the existing DAQs of the centrifuge, with the 12-bit ADC replaced by an 18-bit ADC with support for differential inputs. The I2C cables were shortened as much as possible which also improved reliability significantly. The Raspberry Pi was connected to the Local Area Network (LAN) over ethernet and provided with a static IP address. All the components, fixed using small adhesive pads, were installed in two low cost plastic containers, one on top of the other, with removable lids allowing for ease of access. The two containers were mounted in the instrumentation cabinet with cable ties and connected to the 24 V power supply. A 220 V backup power supply originally installed in the prototype, is also visible in the figure. The conditioned power from the motor controller was wired to the centrifuge’s software-controlled multiplexer and delivered to the model platform on the centrifuge swing from where the actuators were powered. The actuators’ potentiometers were connected to the centrifuge DAQ. For additional flexibility and ease of repair, the ADC was installed onto a small piece of veroboard with screw terminals to accommodate the differential input wires.

### 6.6 Electronic design

Fig. 6 provides a summary of the electrical design along with the respective pin numbers used for the current implementation of the Fly-by-Pi controller. The design schematic is available from the online repository (*FlyByPi_Schematic.pdf*). The motor driver provides the fine level of control required using only three control pins. The direction of motion, sleep and PWM input pins of the motor driver are controlled by three pins on the Raspberry Pi. These three pin controls are exposed in Python as simple software commands for the user that can be combined to create more sophisticated control mechanisms. The original 220 V step down power supply was removed in favour of the centrifuge’s 24 V DC supply that is shared with the motor controller driving the linear actuators. Power for the Raspberry Pi and motor controller logic was supplied by a multi-stage step-down power supply (LM2596), with power to the Raspberry Pi supplied by a standard USB micro-B cable. Depending on the motor type, different motor drivers can be implemented as required. The Raspberry Pi is limited to one hardware PWM pin with software implementations available to provide additional PWM outputs. The signal from the centrifuge’s controller system is linked to the Fly-by-Pi’s ADC input pins using a BNC connection. The calibration factors configured in the centrifuge DAQ system (Volt per metric unit) were stored in the Python files prior to tests.

The combination of complementary spheres of knowledge from STEM (science, technology, engineering and mathematics) was required to realize a successful controller design with the required functionality. This fusion of different fields is collectively referred to as *Civiltronics* [11] within the Department of Civil Engineering at the University of Pretoria, components of which form part of the undergraduate curriculum. The concept of Civiltronics - which remains underpinned by traditional civil engineering – serves to leverage new technologies, delivering innovative solutions. A significant amount of time was dedicated to the initial prototype, with the current implementation of the controller a result of learning from these failures. Whilst it remains time consuming to develop these customized hardware and software solutions, it has proven significantly more cost effective, especially for state-of-the-art research applications.

## 7 Operation instructions

**Fig. 7:**
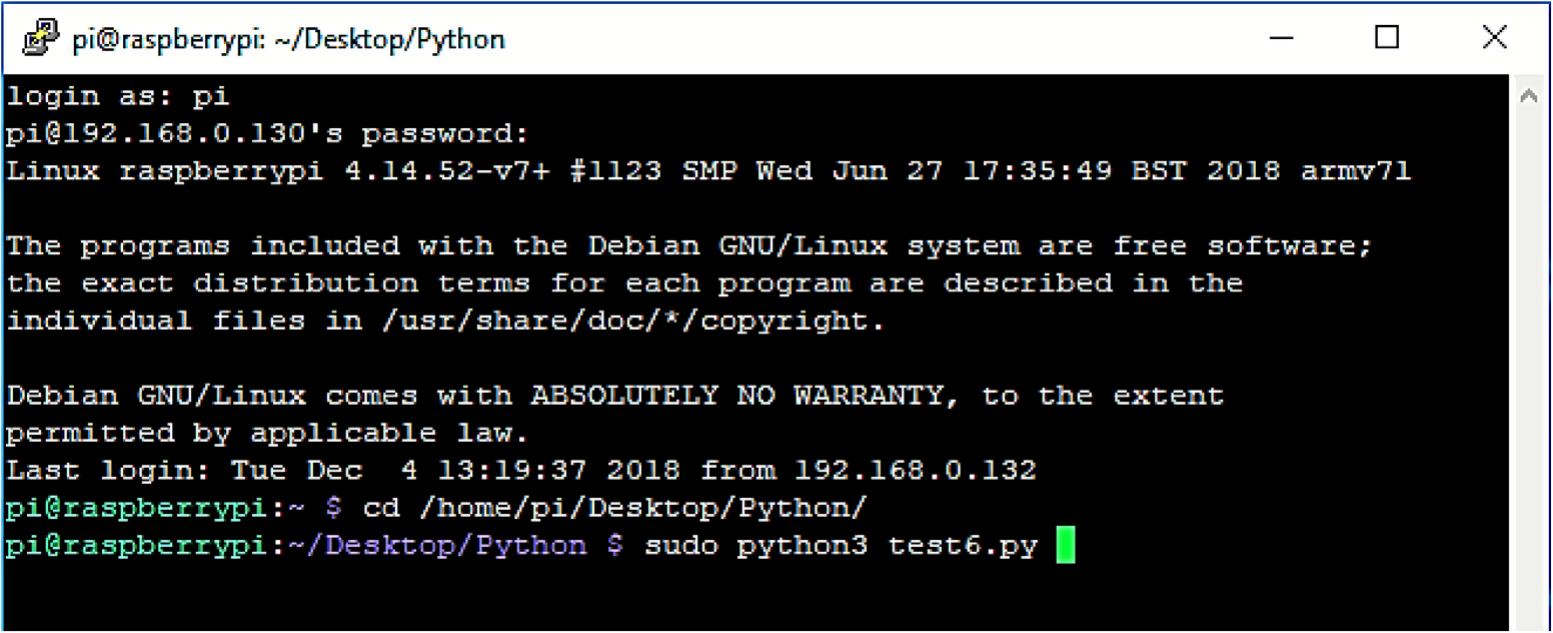
SSH session illustrating the user interface with the Fly-by-Pi controller.

The Fly-by-Pi controller is designed to integrate seamlessly with the existing testing procedures of the centrifuge. The Raspberry Pi is accessed over a secure shell (SSH) terminal using PuTTY with the required software and control scripts preconfigured (Fig. 7). The details of the control scripts are listed in Table 1. In most instances, a script from a previous test was duplicated and adapted for the current experiment using file management and editing tools within the terminal. A dedicated software repository (refer to the *Source Code* folder) is provided on the online repository, along with the user guide. One of three different control mechanisms are implemented for an experiment as discussed in detail below.

### 7.1 Control mechanisms

Three different control mechanisms were implemented during prototyping and experimentation stages. These were:

• Time-based cyclic control
• Load control
• Displacement control

The duty cycle is the main variable controlled in the software in all three control mechanisms. The resulting effect is a variable effective torque available to drive the linear actuator.

*Time-based cyclic control* provides the simplest implementation of actuator control. Push and pull cycles of user defined durations are specified in addition to the duty cycle, which controls the motor torque and hence the maximum exerted force. No auxiliary sensor is used to provide feedback for control of the achieved load or displacement.

**Fig. 8:**
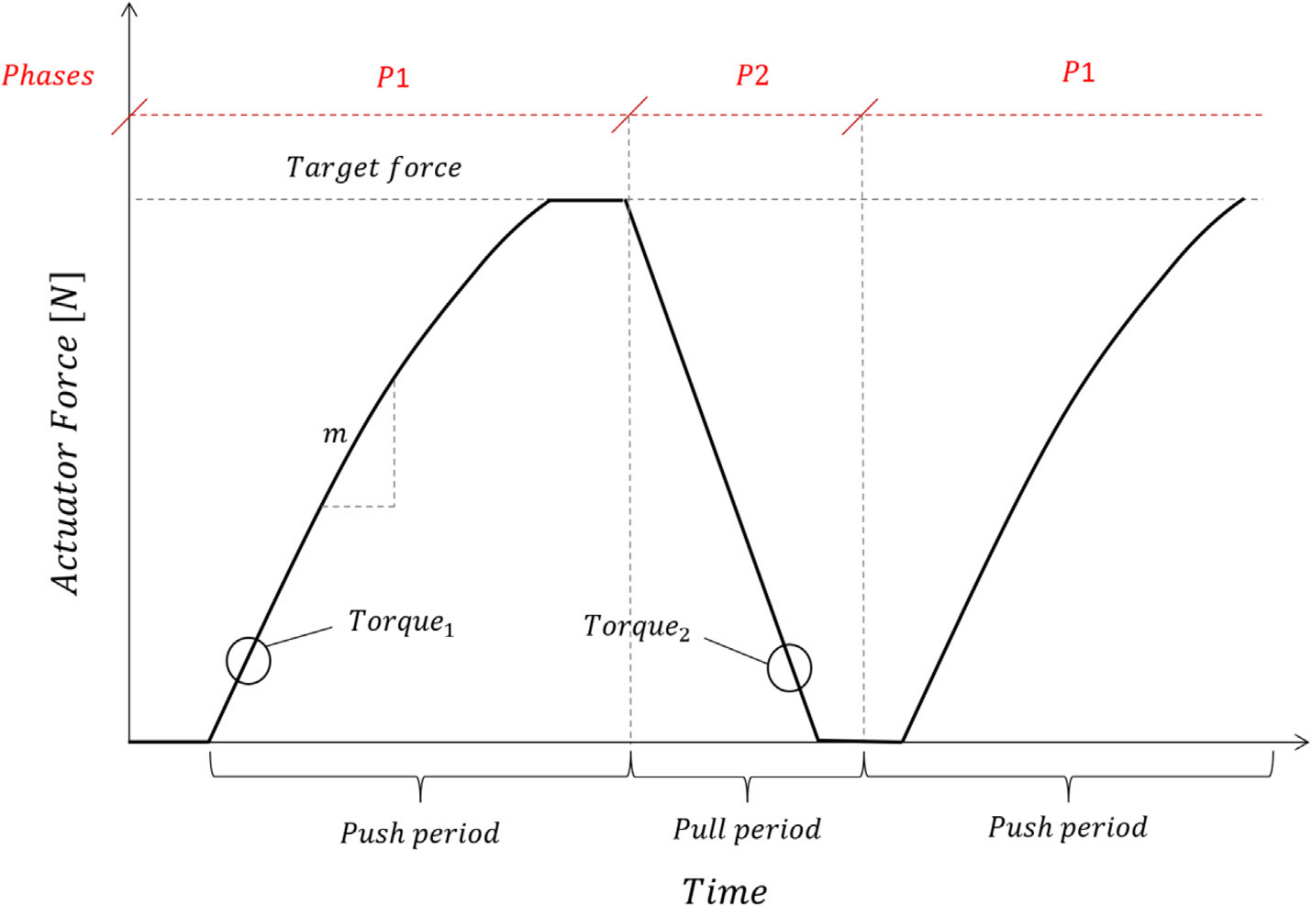
Functional time history and phases for time-based cyclic control applications.

For *time-based cyclic control*, the linear actuator was configured with a constant duty cycle for the duration of the test. A push–pull pattern was followed either indefinitely or for a certain number of cycles. This control mechanism is demonstrated by the sawtooth force–time diagram in Fig. 8. This control mechanism is divided into 2 phases:

• Phase 1: from a retracted position, the actuator is extended using a static duty cycle (Torque_1_) until contact is established with the model. The force increases approximately linearly over most of the phase until the limit of the actuator (as limited by the specified duty cycle) is reached. The actuator then remains idle until phase 2 is initiated to retract the actuator. The total push time (P1) is specified by the user.
• Phase 2: the actuator retracts away from the model with a near instantaneous reduction in force that is linear. The pull time (P2) is specified such that contact is broken between the model interface and the actuator. Note that the specified torque for Phase 2 (Torque_2_) was typically greater than that of Phase 1 to not only increase the effective frequency of the load cycles, but also to take into account permanent deformation caused by the load history.

The application of *time-based cyclic control* resulted in imprecise achievement of the target loads because these were also influenced by the resistance of the model interface against which the actuator was pushing, which changed slowly over many load cycles.

**Fig. 9:**
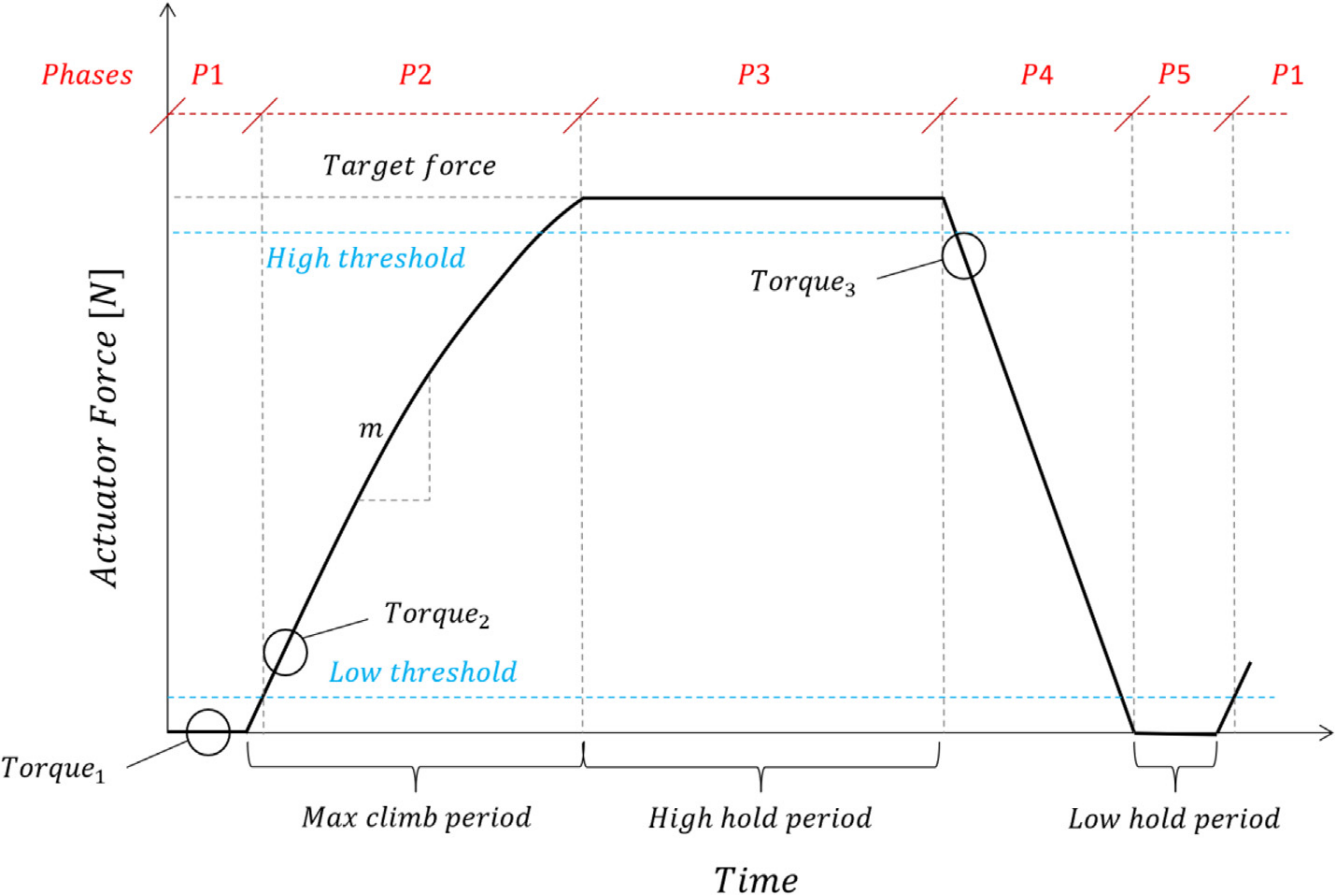
Functional time history and phases for load control applications.

*Load and displacement control* are nearly identical in their implementation. The actuator is limited by either a specified displacement or applied force, as measured by the potentiometer or load cell respectively Fig. 9. Illustrates an idealised load control mechanism, sub-divided into a 5-phase process for each load cycle:

• Phase 1: from a retracted position, the actuator is extended using a static duty cycle (Torque_1_) until contact is established with the model interface. Contact is defined by a lower bound threshold to account for minor variations due to noise and vibration.
• Phase 2: after contact is established, the duty cycle is adjusted to a predefined starting value (Torque_2_). The duty cycle either remains static during this phase or is increased using feedback measurements from the load cell (via the centrifuge’s DAQ). A minimum loading rate threshold can be specified that must be met, failure of which increases the duty cycle incrementally (linearly or exponentially). This works especially well, resulting in consistent loading rates while avoiding stalling the motor. Once an upper bound (higher) threshold is reached, specified as a percentage of the target load, duty cycle gain is disabled to prevent overshoot from delays induced by the feedback loop. If the maximum capacity of the load cell is reached prior to reaching the target load, the timer functionality, initiated at the start of this phase, is flagged to abort the phase and continue onto the next phase. This maximum climb period is specified by the user.
• Phase 3: once the target load is reached, the linear actuator is disabled for a set period. During cyclic loading, this period is usually short so the load reduction due to creep is insignificant.
• Phase 4: the linear actuator is retracted at a specified duty cycle (Torque_3_) until separation of the actuator occurs, again defined by the lower bound threshold based on the load cell measurements.
• Phase 5: movement of the actuator is disabled for a period as specified by the user, after which the next cycle is initiated.

For *displacement control* a nearly identical procedure is followed as with *load control*. The displacement target and upper threshold are specified in terms of displacement measurements. A minimum displacement rate, effectively the velocity of the actuator, dictates the actuator’s duty cycle during phase 2. The lower bound threshold to determine contact of the linear actuator with the model is naturally always specified as a force, regardless of the control mechanism.

From the three control mechanisms presented, the benefits of implementing a programmable control system is clear. The parameters of the control mechanism adopted for an experiment can be adjusted using either static, 1-g tests or in-flight during the experiment involving some trial and error until optimised actuator performance is achieved.

### 7.2 Python implementation

Each control method was implemented as a Python script. A basic framework was developed for the desired control method for ease of implementation by the end uses. During calibration of Fly-by-Pi an average closed-loop control frequency of 63 Hz was achieved. This closed-loop feedback frequency is sufficient to ensure repeatability of the applied loads and avoiding yielding of materials and soils through overshooting the specified target force or displacement. The software makes provision for catching exceptions because of data corruption and electromagnetic interference that was observed periodically during tests above 30-g. With the motor driver pins designed for active high operation, all software scripts automatically implement fail-safe operations; for either intentional or unintentional termination of the script, the motor driver will immediately seize power delivery.

Developmental of the Python scripts was accomplished using PyCharm on a computer in the centrifuge control room and synchronised using FileZilla over the network to the Raspberry Pi on the centrifuge. The users could run their selected scripts with a standard Python 3 environment that is not dependant on 3rd party libraries. Rudimentary object classes were created for both the motor controller and ADC to reduce reliance on external libraries and software updates, given that no internet connectivity is provided to the internal network for security purposes. A User Datagram Protocol (UDP) server-client configuration has been successfully trialled as part of ongoing developments to develop a more user-friendly Graphical User Interface (GUI) for interfacing the Fly-by-Pi to control the system and visualise the information in real-time. The Python implementation thereof can be accessed from the data repository (*UDPDemo* folder). A typical example, using the time-based cyclic control script, is presented below, illustrating the ease of implementation:

# 2020/05/14

# Time-based control @ 30-g

# Researcher: Hendrik Louw

# Test name: lateral pile loading @ 200 N FS

from mMotorDriver import cMotorDriver as md

from time import sleep

motor = md() # create a new actuator instance

motor.setEnable(enabled = 1) # enable the motor

motor.setBackward() # retract the actuator

motor.setSpeed(8) # set duty cycle to 8%

sleep(5) # retract the actuator for 5 s

for cycle in range(1000): # for 1000 cycles

 motor.setForward() # extend the actuator

 print(“Forward”)

 sleep(6) # extend for 6 s

 print(“Cycle no ” + str(cycle + 1))

 motor.setBackward() # retract the actuator

 print(“Backward”)

 sleep(4.75) # retract for 4.75 s

print(“End of test”)

motor.setEnable(enabled = 0) # disable the motor

exit(0) # end of script

## 8 Validation and characterization

Three examples of projects where the Fly-by-Pi controller was implemented are presented.

• The first two examples for which the Fly-by-Pi controller was originally developed involves cyclic loading of single piles in sand using a time-based cyclic controlled actuator, followed by similar tests in an expansive clay using a single load-controlled actuator [15].
• The third example is a study of cave mining where the Fly-by-Pi controller was used as a “smart” power supply, driving five multiplexed linear actuators instead of one.

### 8.1 Laterally loaded piles in sand

As part of a study to investigate the behaviour of piled foundations, a series of cyclic load tests was carried out in the centrifuge, testing both aluminium and reinforced concrete model piles in sand [10], [14] and clay soil profiles. Large numbers of controlled horizontal load cycles had to be applied to the pile heads. The model scale was 1:30, implying that models were tested at 30-g.

**Fig. 10:**
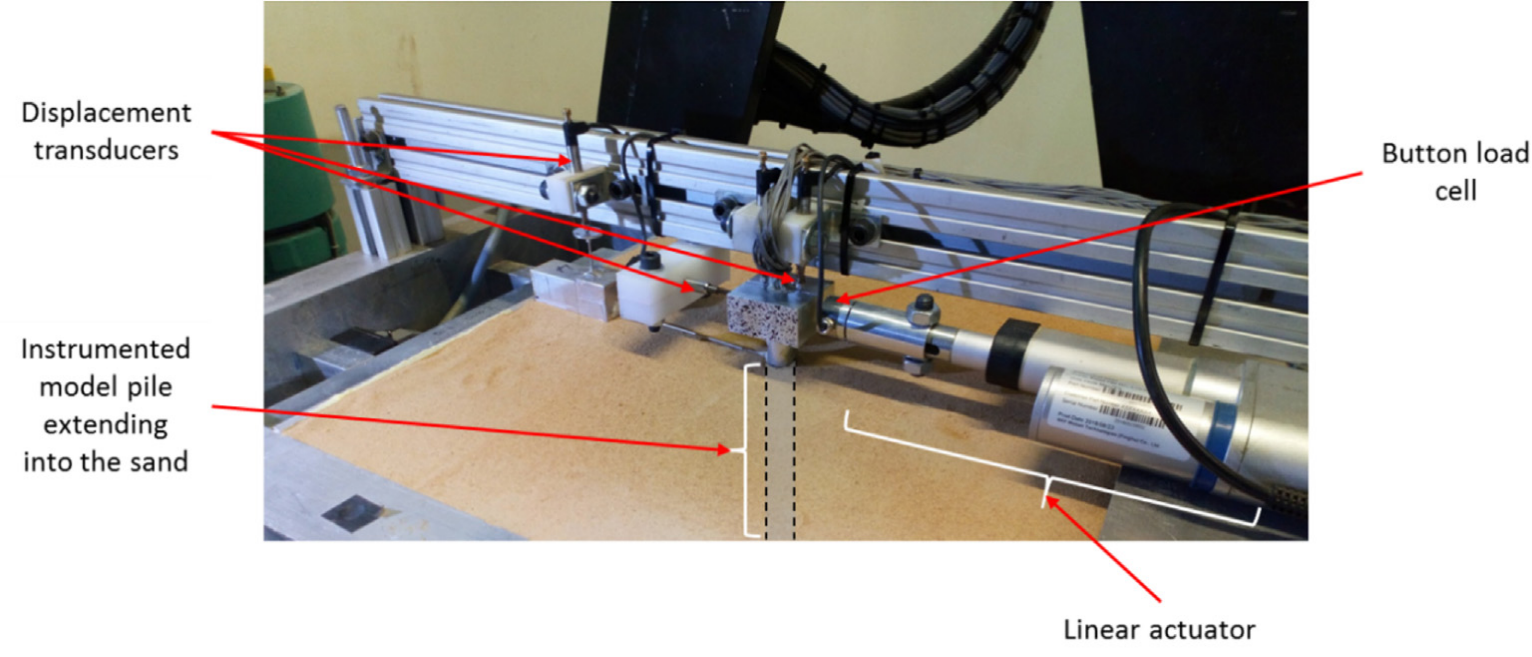
Typical model set-up – laterally loaded piles in sand.

At the time of testing the aluminium and reinforced concrete piles in sand, only the time-based cyclic controlled actuator was developed, which required a constant duty cycle as input, along with a specified cycle duration. Fig. 10 illustrates part of the model showing the aluminium model pile installed in sand. The figure shows an aluminium block (or pile cap), fitted to the pile head, against which load was applied using a SKF CAHB-10 Series linear electric actuator with a stroke of 50 mm. During testing of these piles, the corresponding lateral displacement was measured using a number of displacement transducers above and below the soil surface, with the bending moment development in the piles measured using strain gauges that were attached to the sides of each pile. The magnitude of the applied load was measured using a button load cell attached to the front of the linear electric actuator shaft.

**Fig. 11:**
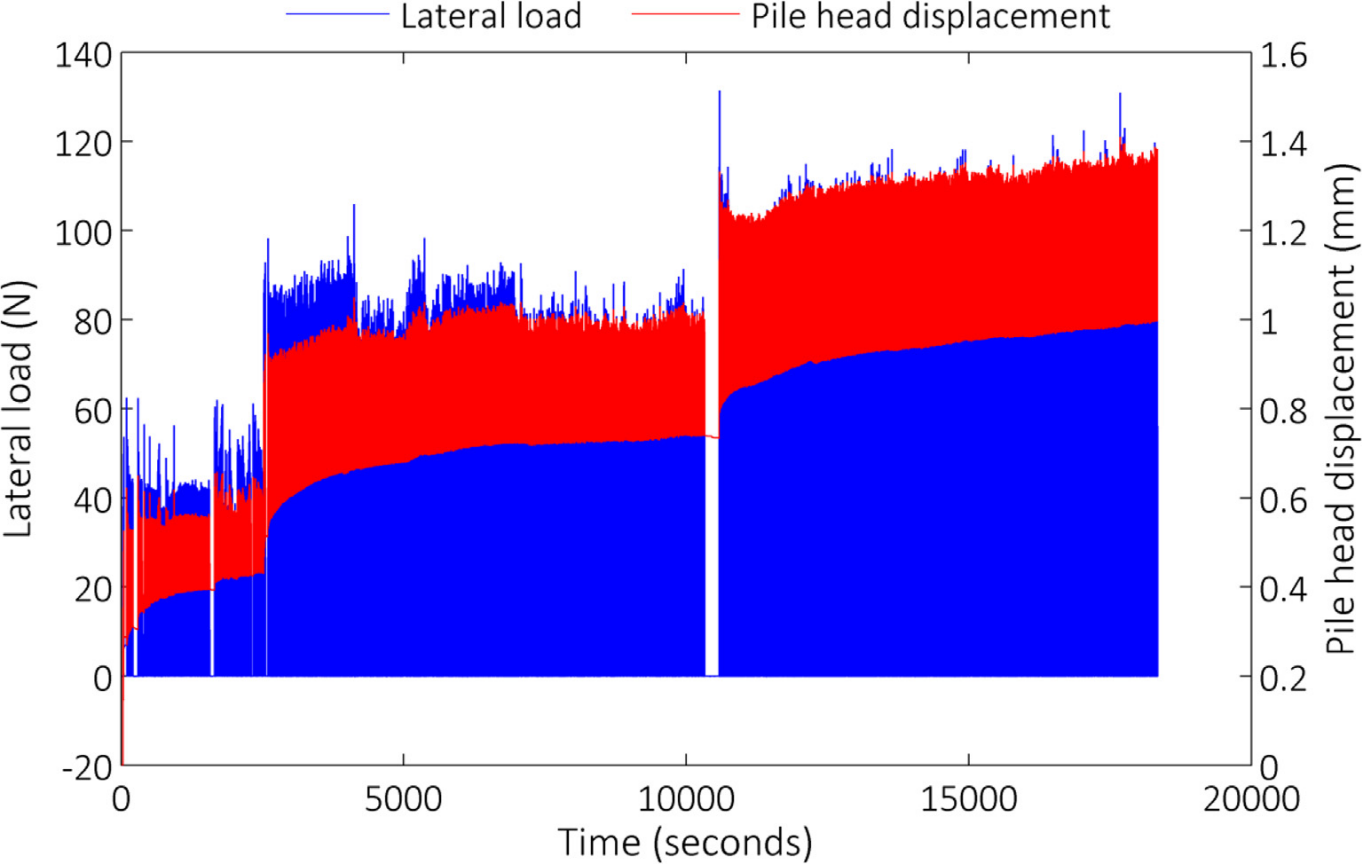
Lateral load/pile head response – aluminium model pile.

**Fig. 12:**
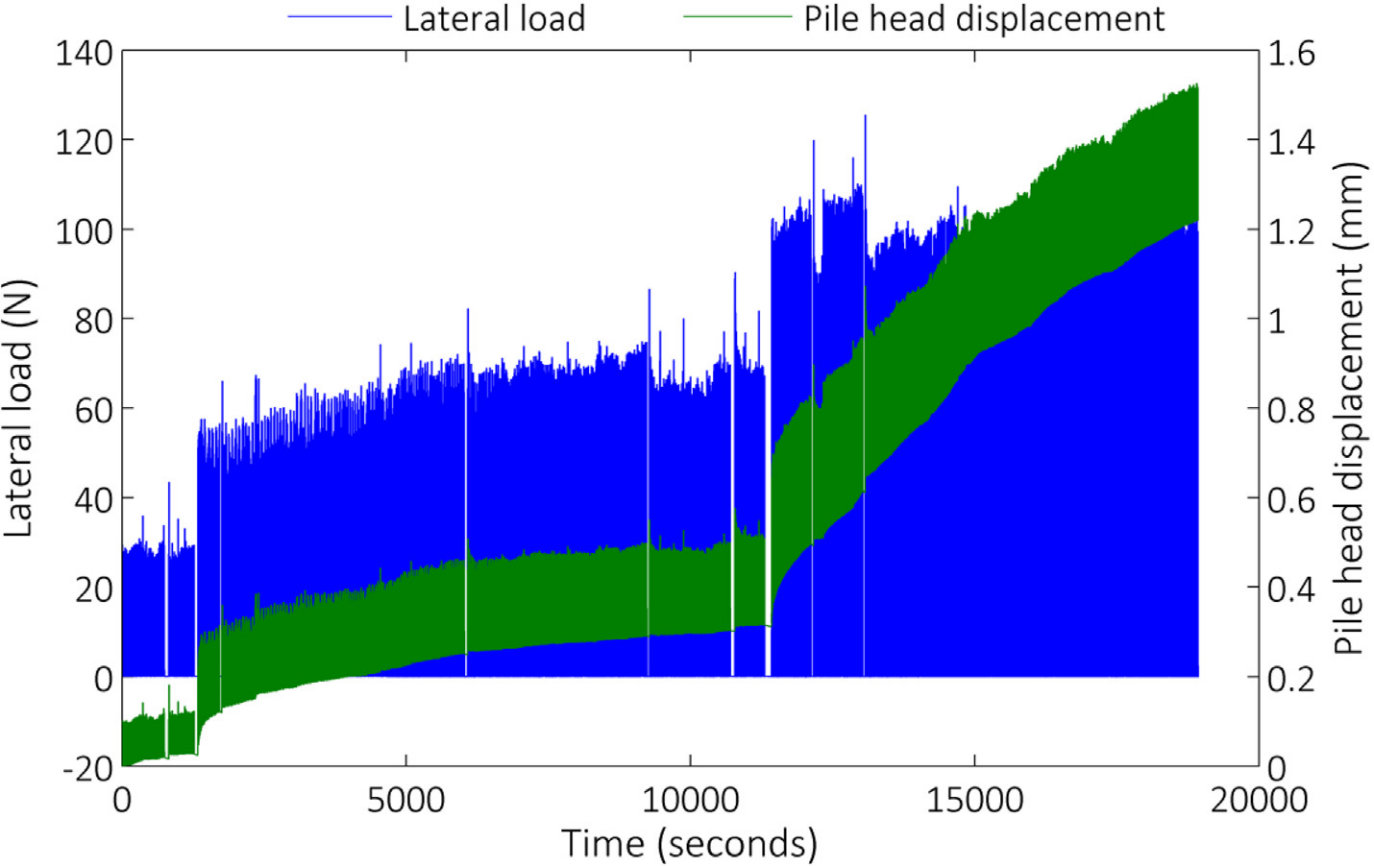
Lateral load/pile head displacement response – reinforced concrete model pile in sand.

Testing involved several thousand load applications to a certain load magnitude, after which the target load was increased and the load cycles repeated. Up to three load magnitudes were thus applied. Fig. 11 and Fig. 12 indicate the lateral load response on the primary vertical axis for both the aluminium and reinforced concrete model piles, respectively, for the duration of each test. In addition, the corresponding pile head displacement is shown on the secondary vertical axis in both figures. From the load response it can be seen that total load control was not possible, mainly due to the fact that cycle duration had to be specified, which had to be updated regularly as the tests progressed as a result of the previous load cycles, which affected the position of the pile. Regardless, in-flight adjustment of the control mechanism still proved invaluable during testing.

Vertical cyclic load tests were later repeated on the aluminium and reinforced concrete model piles in an expansive clay using a load-controlled actuator.

**Fig. 13:**
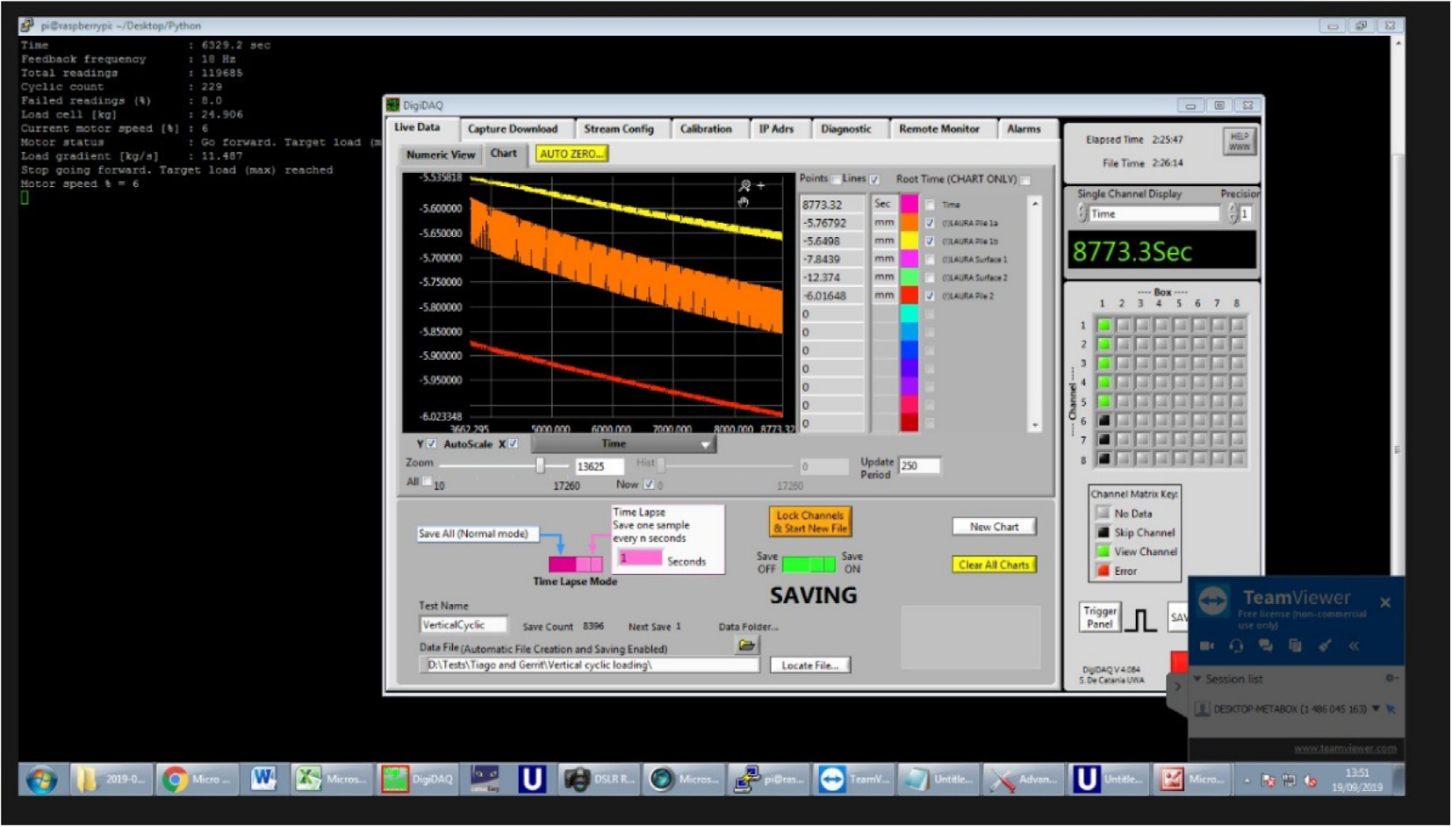
Remote centrifuge management over the internet.

For tests that ran over a longer period spanning days, adjustment of the software scripts was performed remotely using TeamViewer™ (Fig. 13). This also proved valuable to provide tele-assistance during the preparation and installation of new experiments in the centrifuge.

### 8.2 Vertically loaded piles in clay

**Fig. 14:**
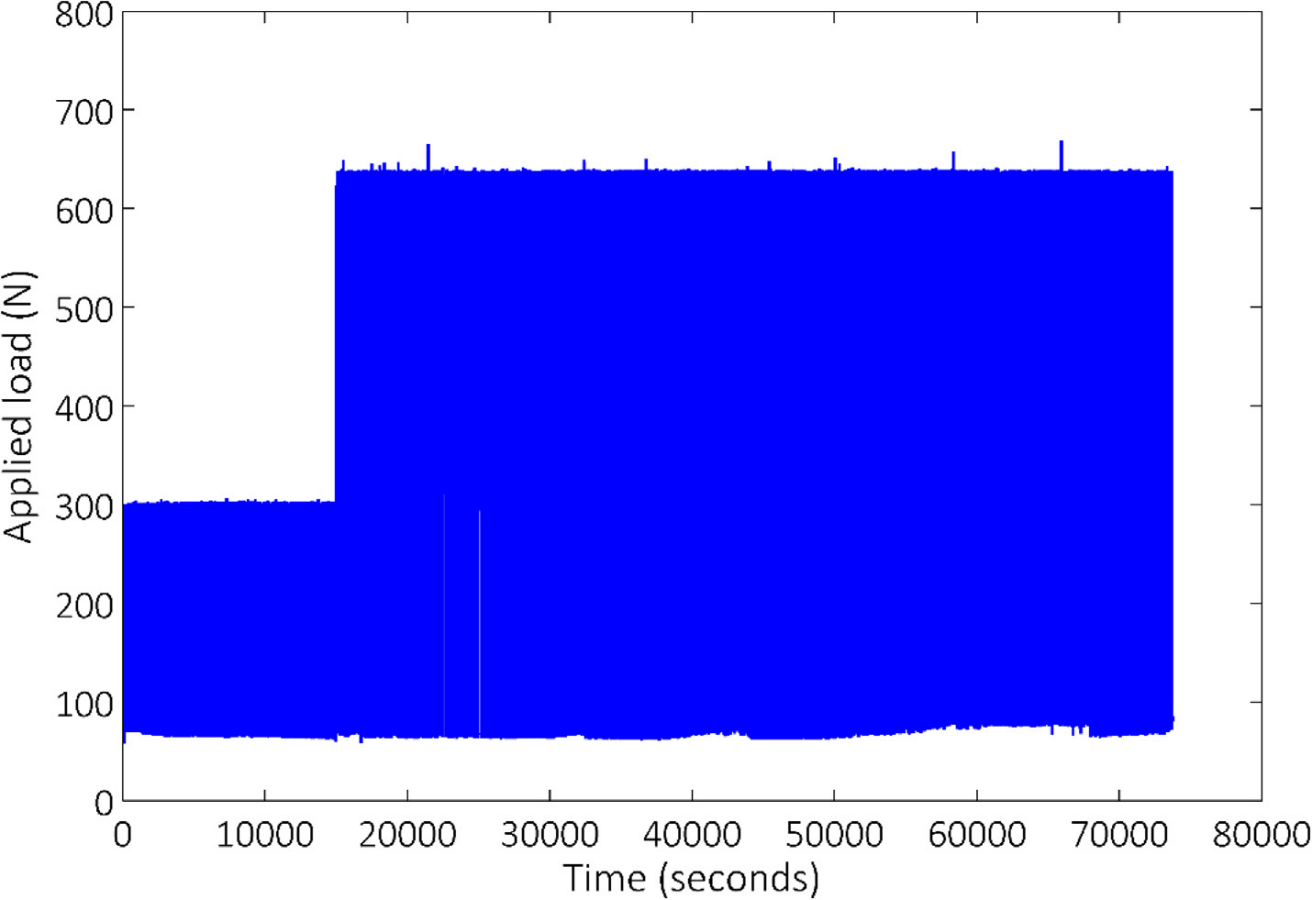
Illustration of the performance of the Fly-by-Pi controller for a long-term test in a stiff clay at natural moisture content showing near-perfect load control.

**Fig. 15:**
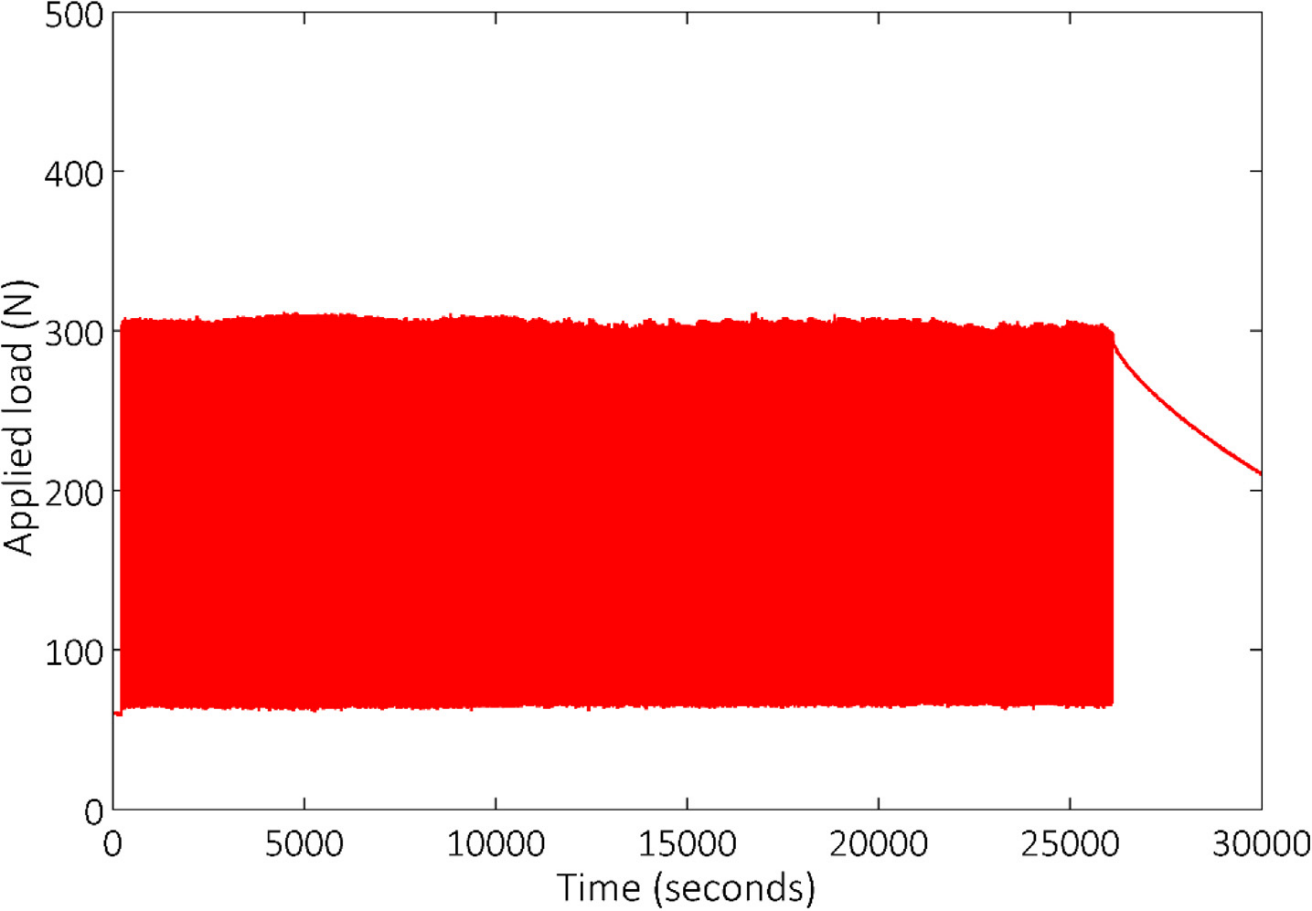
Illustration of the performance of the Fly-by-Pi controller for a long-term test in a stiff clay whilst the clay was undergoing swelling.

A significant property which differentiates soils from other materials are that their stiffness is significantly strain dependent [25] i.e. the more strain experienced by the material, the less stiff it becomes. This issue is further exacerbated when considering swelling clays. Such clays exhibit volume change upon wetting as well as swell induced softening [26]. These combined effects increase the sophistication required for a load control system. Despite these constraints, Fig. 14 illustrates almost perfect load control during the cyclic loading of a concrete pile in stiff clay at its natural moisture content. An arguably more impressive illustration of the load control system is the result presented in Fig. 15. Throughout the time period presented, the clay was undergoing a swelling process and so there was a reduction in soil stiffness as the test progressed. Despite the continual change in soil stiffness, the developed load-control system could maintain the targeted load for the required number of cycles.

The statistics presented in Table 4 summarises the quantitative performance of the Fly-by-Pi controller. These statistics refer to clay sample that was tested at both the natural moisture content using a maximum applied load of 300 N and 600 N (Fig. 14) in addition to the swelling sample tested with a maximum load of 300 N (Fig. 15). The maximum load for every load cycle was determined using a Python script which identifies all such samples for the entire dataset. A total of 1322 load cycles were applied for the natural moisture content samples (535 and 787 cycles for a target load cycle of 300 N and 600 N respectively) and 967 load cycles for the selling clay sample. The average peak load measured by the load cell correspond to the target load with only a small standard deviation. For the target load of 600 N, the average load was higher that the target load, but still remained within a narrow operating envelope. If the maximum load is considered for a single set consisting of two consecutive load cycles, the difference of this set should ideally be small. This is reflected in the statistics; for the 300 N target load a standard deviation of 0.6 N can be expected, compared to approximately 2.2 N for a 600 N target load for a given set.

**Table 4:** Performance statistics of the Fly-by-Pi controller.

Test description	Statistics of the applied load [N]			
	Minimum	Average	Maximum	Standard Deviation
All maximum peak loads	All maximum peak loads			
Natural moisture (300 N target load)	300.0	301.9	304.7	0.679
Natural moisture (600 N target load)	637.5	638.9	663.9	1.756
Swelling (300 N target load)	300.8	306.5	311.1	2.207
	Load difference of a set			
Natural moisture (300 N target load)	−2.160	0.003434	2.200	0.5934
Natural moisture (600 N target load)	−25.38	0.0003794	21.46	2.228
Swelling (300 N target load)	−2.403	−0.001087	2.979	0.5927

**Fig. 16:**
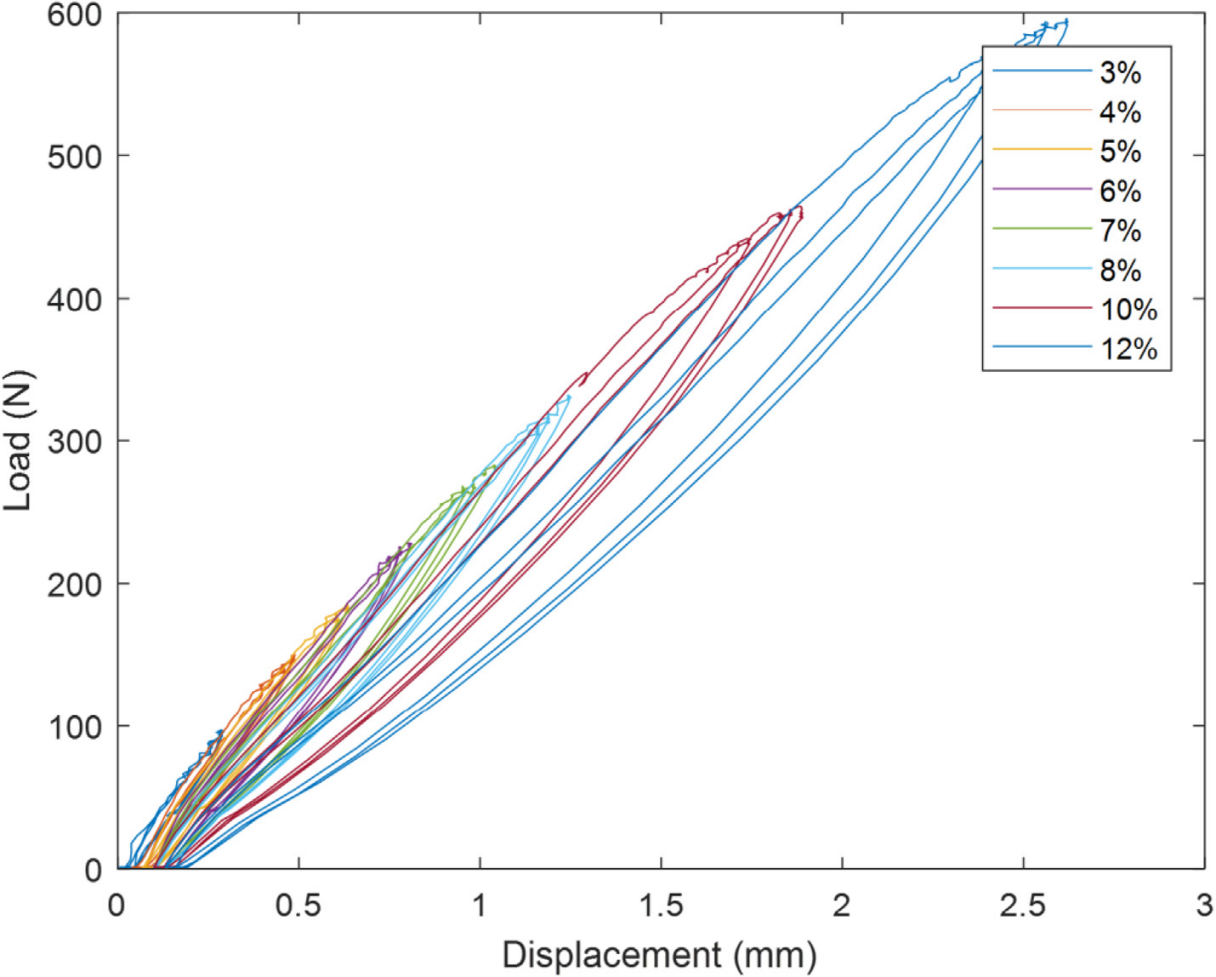
Load displacement cycles for 3 cycles at increasing motor powers (duty cycles).

**Fig. 17:**
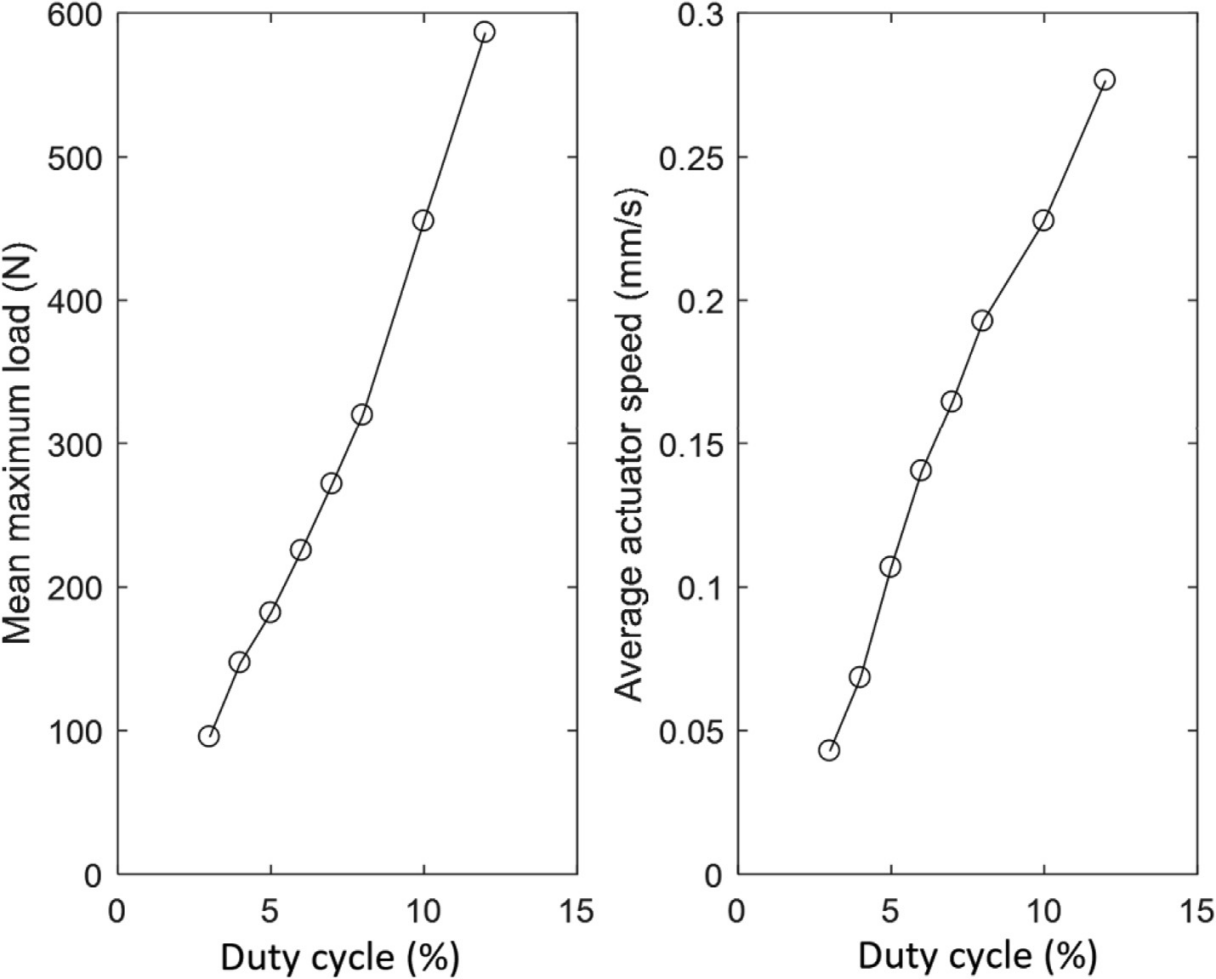
Relationship between duty cycle, the maximum load and average actuator motor speed.

The load–displacement curves for three cycles at increasing motor powers are shown in Fig. 16. The maximum load in each cycle was determined; the average of these was plotted as a function of the motor power (duty cycle) in Fig. 17.

### 8.3 Cave mining study

Cave mining involves the undermining of an ore body, removing support in a controlled way and allowing the rock mass to fracture under its own weight. It is an efficient mining operation as it minimises the need for blasting. The condition of the rock mass being fractured is assessed by acoustic monitoring calibrated against numerical models. However, it is rarely possible to verify predictions and analysis results because access to the rock mass is not possible. By carrying out model studies of the cave mining process, it is hoped to learn more about this mining method.

A centrifuge model was developed to study cave mining in the geotechnical centrifuge [27]. The model comprised of a weakly cemented artificial rock mass, supported in a stiffened aluminium frame and viewed through a 30 mm thick glass window. Five 50 mm wide trapdoors, supported by five SKF CAHB-10 Series linear electric actuators each with a stroke of 100 mm, were used to remove support from the artificial rock mass in a controlled way to observe the resulting fracture mechanism. Acoustic emission sensors were used to track fracture events and compare against visual observations. The tests were carried out in the centrifuge at 80-g.

For this project, the Fly-by-Pi controller was used as single software-adjustable power supply to drive all five actuators. The conditioned power output from the Fly-by-Pi controller was fed through the centrifuge’s programmable multiplexer. This allowed the actuators to be lowered at a precisely controllable rate in a sequence managed via the multiplexer by means of a pre-programmed sequence or by manually switching the actuators on and off. The ability to terminate the pre-programmed sequence and switch to manual control allowed for useful versatility to respond to events in the caving sequence. The rate of lowering was controlled by setting an appropriate duty cycle. In addition, the direction of the trapdoors could be reversed by a single minor adjustment to the software during testing. This application illustrates versatility of the Fly-by-Pi system.

**Fig. 18:**
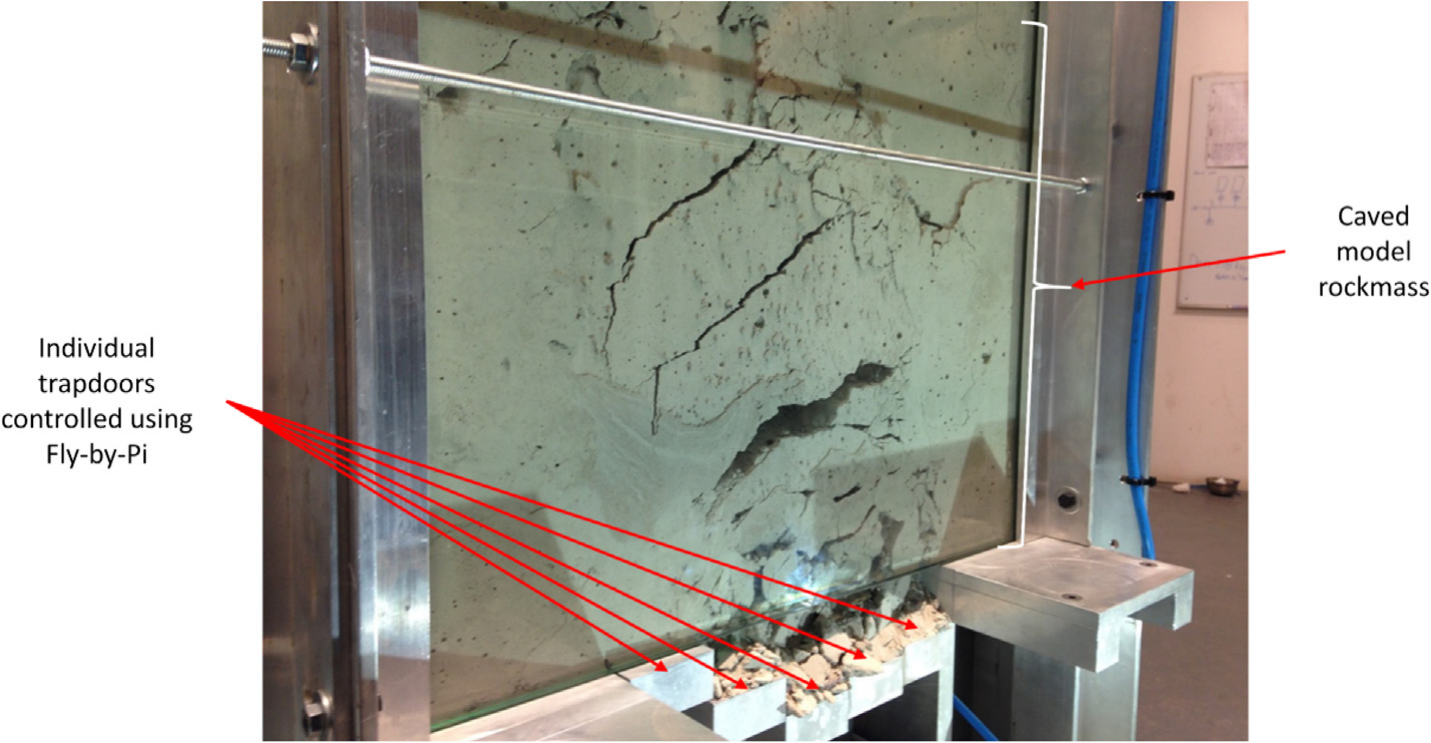
Centrifuge model demonstrating a caved model rock mass, also showing the individual trapdoor below controlled using the Fly-by-Pi controller.

**Fig. 19:**
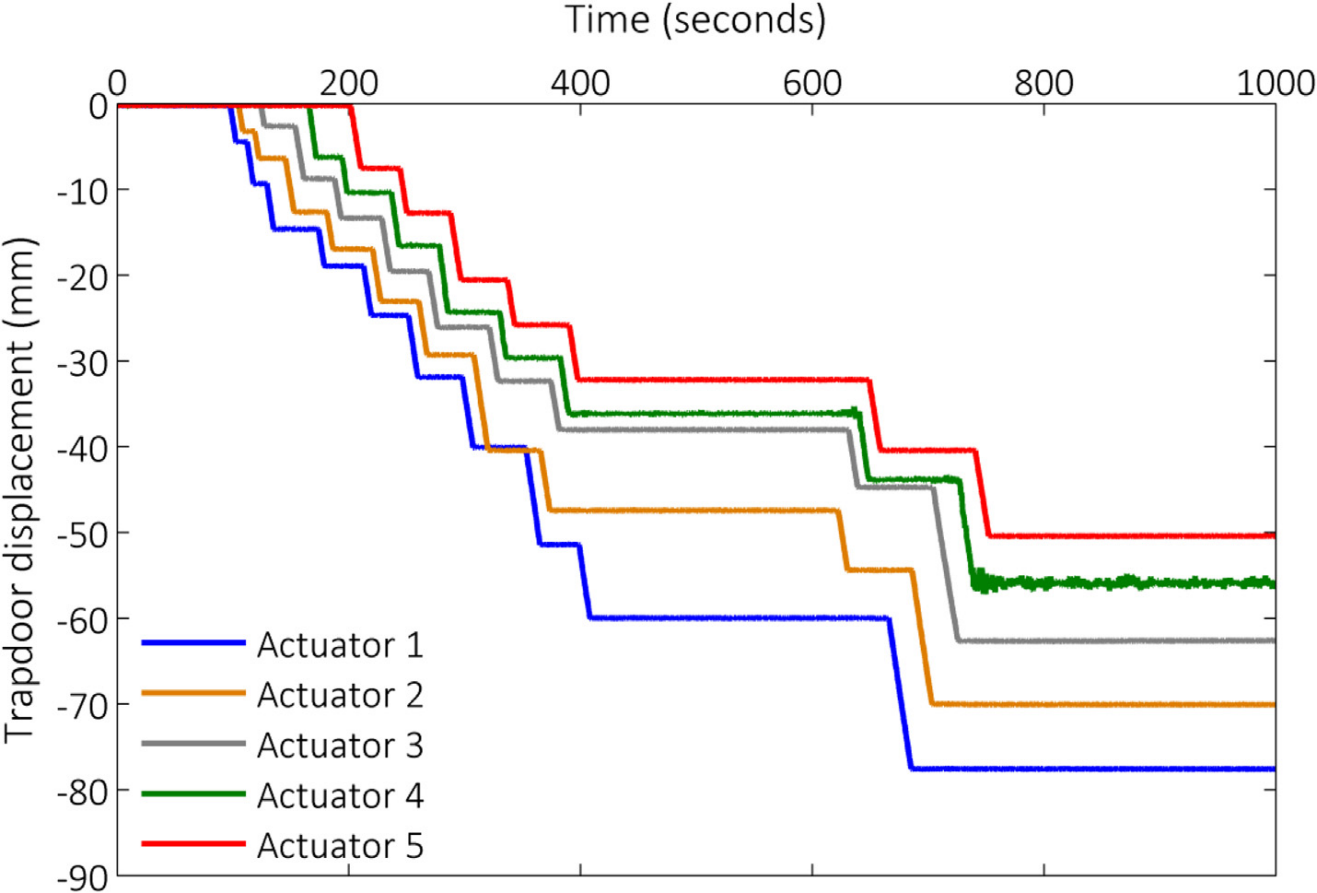
Trapdoor displacements achieved using automated and manual control, powered by the Fly-by-Pi controller.

Fig. 18 shows a centrifuge model after a test, illustrating the fractured model rockmass which resulted from the controlled lowering of the five trapdoors (also highlighted in the figure). The associated recorded actuator displacements, monitored using potentiometers incorporated in the SKF actuators, are illustrated in Fig. 19.

Based on the experimental results and experience gained from the three tests, the following capabilities and limitations of the Fly-by-Pi controller are summarised:

• The Fly-by-Pi controller is suitable for a wide variety of linear actuators and motors. For this implementation, a maximum axial force of 600 N was achieved.
• Fine control over the load application can be accomplished by varying the duty cycle, from 0% to 100%. The resolution thereof is however limited for smaller duty cycles.
• A relatively high closed-loop feedback frequency of 63 Hz was achieved which is sufficient for low-frequency load applications. This can be improved further by implementing a high-performance ADC.
• Multiple linear actuators or motors can be controlled independently through the use of a simple, low-cost relay multiplexer using a single Fly-by-Pi controller.
• The motor driver operates in a fail-safe manner; whenever a software script is terminated or power is lost to the system, the motor driver reverts to an idle state.
• Full software control is maintained even when the centrifuge is in flight, providing flexibility for altering and modifying parameters during an experiment.
• Repeatable load control was achieved for even highly variable materials such as swelling clay and sand.
• Versatile and complex control mechanisms successfully addressed the need for different experimental requirements.
• The Fly-by-Pi controller proved reliable when subjected to prolonged periods of accelerations, with little to no electrical interference from the adjacent centrifuge electrical motors.
• Although the Raspberry Pi is typically considered consumer grade hardware, no hardware failure has been experienced to date despite the harsh operating environment.

## Funding

This research did not receive any specific grant from funding agencies in the public, commercial, or not-for-profit sectors.

## CRediT authorship contribution statement

**André Broekman:** Conceptualization, Data curation, Formal analysis, Investigation, Methodology, Software, Validation, Writing - original draft. **Schalk Willem Jacobsz:** Funding acquisition, Project administration, Resources, Supervision, Writing - review & editing. **Hendrik Louw:** Formal analysis, Investigation, Methodology, Validation, Visualization. **Elsabé Kearsley:** Funding acquisition, Project administration, Resources, Supervision. **Tiago Gaspar:** Formal analysis, Investigation, Writing - review & editing. **Talia Simone Da Silva Burke:** Formal analysis, Data curation, Investigation, Validation.

## Declaration of Competing Interest

The authors declare that they have no known competing financial interests or personal relationships that could have appeared to influence the work reported in this paper.
